# From Bench-Top to Bedside: A Prospective *In Vitro* Antibiotic Combination Testing (*i*ACT) Service to Guide the Selection of Rationally Optimized Antimicrobial Combinations against Extensively Drug Resistant (XDR) Gram Negative Bacteria (GNB)

**DOI:** 10.1371/journal.pone.0158740

**Published:** 2016-07-21

**Authors:** Yiying Cai, Nathalie Grace Chua, Tze-Peng Lim, Jocelyn Qi-Min Teo, Winnie Lee, Asok Kurup, Tse-Hsien Koh, Thuan-Tong Tan, Andrea L. Kwa

**Affiliations:** 1 Department of Pharmacy, Singapore General Hospital, Singapore, Singapore; 2 Department of Pharmacy, Faculty of Science, National University of Singapore, Singapore, Singapore; 3 SingHealth Duke-NUS Medicine Academic Clinical Programme, Duke-NUS Medical School, Singapore, Singapore; 4 Infectious Diseases Care, Mount Elizabeth Hospital, Singapore, Singapore; 5 Department of Microbiology, Singapore General Hospital, Singapore, Singapore; 6 Department of Infectious Diseases, Singapore General Hospital, Singapore, Singapore; 7 Emerging Infectious Diseases, Duke-NUS Medical School, Singapore, Singapore; Lee Kong Chian School of Medicine, SINGAPORE

## Abstract

**Introduction:**

Combination therapy is increasingly utilized against extensively-drug resistant (XDR) Gram negative bacteria (GNB). However, choosing a combination can be problematic as effective combinations are often strain-specific. An *in vitro* antibiotic combination testing (*i*ACT) service, aimed to guide the selection of individualized and rationally optimized combination regimens within 48 hours, was developed. We described the role and feasibility of the *i*ACT service in guiding individualized antibiotic combination selection in patients with XDR-GNB infections.

**Methods:**

A retrospective case review was performed in two Singapore hospitals from April 2009–June 2014. All patients with XDR-GNB and antibiotic regimen guided by *i*ACT for clinical management were included. The feasibility and role of the prospective *i*ACT service was evaluated. The following patient outcomes were described: (i) 30-day in-hospital all-cause and infection-related mortality, (ii) clinical response, and (iii) microbiological eradication in patients with bloodstream infections.

**Results:**

From 2009–2014, the *i*ACT service was requested by Infectious Disease physicians for 39 cases (20 *P*. *aeruginosa*, 13 *A*. *baumannii* and 6 *K*. *pneumoniae*). Bloodstream infection was the predominant infection (36%), followed by pneumonia (31%). All *i*ACT recommendations were provided within 48h from request for the service. Prior to *i*ACT-guided therapy, most cases were prescribed combination antibiotics empirically (90%). Changes in the empiric antibiotic regimens were recommended in 21 (54%) cases; in 14 (36%) cases, changes were recommended as the empiric regimens were found to be non-bactericidal *in vitro*. In 7 (18%) cases, the number of antibiotics used in combination empirically was reduced by the *i*ACT service. Overall, low 30-day infection-related mortality (15%) and high clinical response (82%) were observed. Microbiological eradication was observed in 79% of all bloodstream infections.

**Conclusions:**

The *i*ACT service can be feasibly employed to guide the timely selection of rationally optimized combination regimens, and played a role in reducing indiscreet antibiotic use.

## Introduction

The emergence of extensively-drug resistant (XDR) Gram negative bacteria (GNB) is increasingly recognized as one of the greatest threat to healthcare [1]. Infections caused by these organisms have reported mortality rates of over 50%, and have been associated with increased healthcare costs [2, 3]. This is worsened by the fact that the antibiotic pipeline against GNB has dried out. Consequently, physicians are compelled to resort to ‘old’ antibiotics such as the polymyxins for the treatment of these infections [1, 4].

In the recent years, there have been an increasing number of physicians employing combination antibiotic therapy against XDR-GNB, in particularly against *Pseudomonas aeruginosa*, *Acinetobacter baumannii* and *Klebsiella pneumoniae* [5]. The contentions supporting combination antibiotics are aplenty. When two or more antibiotics are combined, there may be potential additive or even synergistic activity. In addition, combination antibiotics may suppress the emergence of resistant subpopulations, which can otherwise be selectively amplified if only one antibiotic was used [6]. On the flipside, irrational use of antibiotic combinations can worsen the already alarming scenario of antimicrobial resistance [6]. This has led experts to conclude that if combination antibiotics are employed, the most appropriate combination scheme should leverage on the latest *in vitro* and/or animal infection models, and should be individualized depending on organism, susceptibility profile, infection site and the clinical characteristics of each patient [5, 7].

In view of the current antimicrobial landscape, a prospective *in vitro* antibiotic combination testing (*i*ACT) service, with a rapid turn-around time of 48 hours, was developed. The objective of the *i*ACT service is to guide physicians in the selection of an individualized and rationally optimized combination therapy, taking into account the *in vitro* combination testing results of each strain and the patient’s clinical and pharmacokinetic (PK) parameters. In this study, we described the role and feasibility of the *i*ACT service in guiding the selection of individualized and rationally optimized combinations in patients with XDR-GNB infections. In addition, the clinical and microbiological outcomes of patients receiving *i*ACT-guided therapy were described.

## Materials and Methods

### Study Design

A retrospective case review was conducted in two Singapore hospitals: Singapore General Hospital, a 1700-bed public hospital and Mount Elizabeth Hospital, a 272-bed private hospital. All adults admitted from 1^st^ April 2009 to 30^th^ June 2014 with microbiologic evidence of XDR-GNB or pan-drug resistant (PDR) GNB isolation causing clinical infections and treated with antibiotic regimen guided by *i*ACT were identified from the service’s electronic database and retrospectively reviewed. The use of *i*ACT to guide antibiotic combination selection in these patients was elected by their attending ID physicians as part of their clinical management strategy, out of desperate measures. Patients with infections due to an XDR-/PDR-GNB requiring *i*ACT-guided therapy that were classified as cured, and then developed a subsequent infection after a 90-day period due to a different GNB requiring *i*ACT-guided therapy were analyzed as two different cases. This study was approved by the ethics review board of Singapore General Hospital (2012/818/D) and Mount Elizabeth Hospital (PIEC/2012/038) prior to study initiation. The ethics review board of both institutions waived the need for informed consent as the study involved only a retrospective clinical notes review, and all data collected was analyzed anonymously.

### Description of the Prospective *i*ACT Service Workflow

Before the implementation of the *i*ACT service, the service was introduced to the Infectious Diseases (ID) physicians in the hospitals, to increase awareness of and provide education about the service. Prior to request of the *i*ACT service for a patient with an infection caused by an XDR- or PDR-GNB isolate, the attending ID physician informed each patient (or immediate family member if the patient was incapacitated) of (i) the poor prognostic outcomes of such resistant infections which most or all antibiotics were not effective against, (ii) the nature of the *in vitro* testing method as a last-resort measure to guide treatment, (iii) alternative treatment strategies (e.g. use of polymyxin monotherapy or selecting combinations based on anecdotal experience), iv) the clinical aims of the *i*ACT guided treatment, and (v) the potential toxicities of the treatment. Verbal consent was sought and documented in clinical notes by the attending ID physician before *i*ACT was requested.

**Fig 1:**
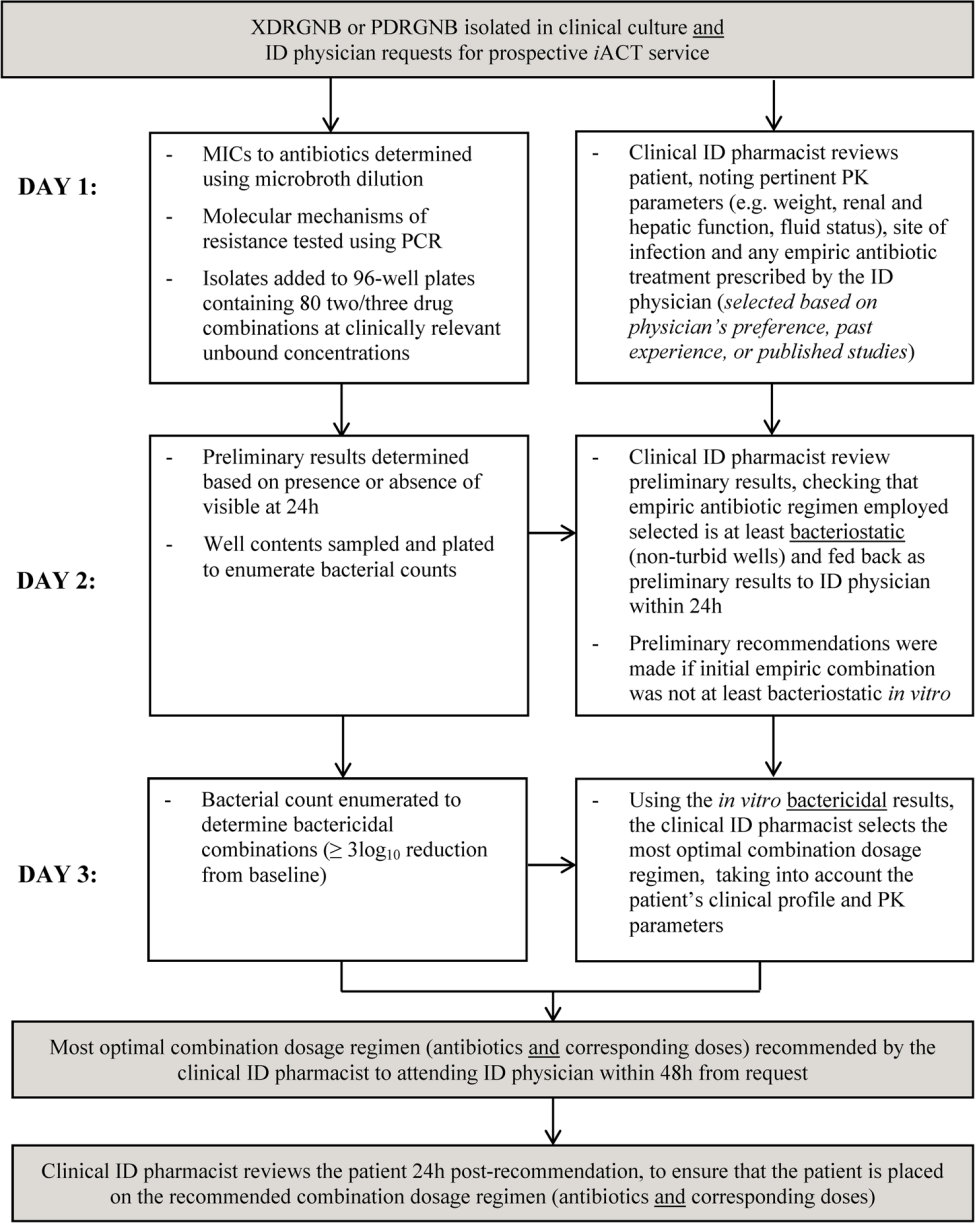
Summary of the Work Process of the Prospective *i*ACT Service. Combination antibiotic regimens recommended by the *i*ACT service take into account both the *in vitro* bactericidal activity of the combinations and the probability of PK/PD target attainment, and are recommended to the attending ID physician within 48h from request. Abbreviations used in Fig 1: GNB = Gram-negative bacteria, *i*ACT = *in vitro* antibiotic combination testing, ID = Infectious Diseases, MIC = minimum inhibitory concentration PDR = pan-drug resistant, PK = pharmacokinetic, XDR = extensively-drug resistant.

The process of the *i*ACT service is detailed in Fig 1. *In vitro* combination testing was carried out immediately upon request of *i*ACT for an individual patient. Simultaneously, a clinical ID pharmacist would review the patient’s clinical records and document all relevant clinical information required to develop rationally optimized combination dosage regimens, based on pharmacokinetics (PK) and pharmacodynamics (PD) principles. At 24h, combinations that were at least bacteriostatic (non-cloudy wells) *in vitro* were identified and fed back as preliminary results to the ID pharmacist. Preliminary recommendations to change antibiotic combinations were made by the ID pharmacist to the attending ID physician, if the initial empiric regimen was not at least bacteriostatic *in vitro*. At 48h, combinations with bactericidal activity *in vitro* were elucidated. Based on these *in vitro* results, the ID pharmacist would select and recommend the most optimal combination(s), with corresponding dosage regimens, to the attending ID physician. Selection was based on the patient’s clinical characteristics, site of infection, PK/PD parameter for efficacy of each antibiotic and the probabilities of PK/PD target attainment at infected sites. Clinical ID pharmacist followed-up and reviewed the patient’s case notes within 24h post-recommendation, to ensure that the patient was placed on the recommended antibiotic combination regimens.

### Microbiological Methods

Genus identity was determined using VITEK GNI+ cards (bioMérieux, Hazelwood, MO). MICs to various antibiotics were determined using commercial microbroth dilution panels (Trek Diagnostics, UK), and susceptibility was defined as per CLSI [8]. All *A*. *baumannii* isolates were screened for *bla*_OXA-23-like_, *bla*_OXA-24-like_, *bla*_OXA-51-like_, and *bla*_OXA-58-like_ genes using a multiplex PCR assay [9]. For *P*. *aeruginosa* and *K*. *pneumoniae* isolates, PCR was employed to detect genes encoding commonly acquired metallo-β-lactamases (MBLs) (*bla*_VIM_, *bla*_IMP_, *bla*_SIM_, *bla*_GIM_, *bla*_SPM_) [10, 11]. Analyses of genes encoding ESBLs, AmpC, OXA, KPC and NDM beta-lactamases were also performed for *K*. *pneumoniae* [10, 11]. Changes in porin gene expression (OmpK35 and OmpK36) were determined for *K*. *pneumoniae* using RT-PCR, and presence of efflux pump was determined using efflux pump inhibitor phenyl-arginine-β-naphthylamide (PABN) [8, 12].

*In vitro* combination testing was carried out in clear 96-well microtiter plates, modified from a method by Aaron *et al* [13]. Briefly, 100uL of bacterial inoculum was added to a microtiter plate containing 11 antibiotics in single, two-drug and three-drug combinations (100uL of antibiotic solution per well) at clinically relevant unbound concentrations (Table 1) [14–25]. This gave approximately 10^5^ CFU/mL (1 × 10^5^–5 × 10^5^ CFU/mL) of bacteria in each well. The wells were covered and incubated at 35°C for 24h. At 24h, bacteriostatic combinations were identified based on presence/absence of visible growth in each well. The contents of each well were then sampled and measured to ensure <10% loss in volume. The measured contents were centrifuged at 10,000 × g for 15 minutes and the pellet reconstituted to original volume to minimize drug carry-over. Bacterial count was quantified by depositing serial ten-fold dilutions of the sample onto Mueller-Hinton agar plates (BD, Sparks, MD), incubated at 35°C for 24h, and enumerated visually to determine presence of bactericidal activity. The lower limit of detection was 3.9 × 10^2^ CFU/ml.

**Table 1:** Simulated Antibiotic Dosing Regimens and Corresponding Drug Concentrations.

Drugs	Site of Infection Simulated	Simulated Dosing Regimens	Concentration (mg/L)
Amikacin [14]	Plasma	20 mg/kg every 24h	65
Levofloxacin [15]	Plasma	750mg every 24h	8
Levofloxacin [16]	Pulmonary ELF	750mg every 24h	20
Rifampicin [17]	Plasma	600mg every 12h	4
Polymyxin B [18]	Plasma	30,000IU/kg/day	2
Tigecycline [19]	Tissue	100mg every 12h	2
Cefepime [20]	Plasma	2g every 8h (infused over 4h)	50
Meropenem [21]	Plasma	2g every 8h (infused over 3h)	20
Doripenem [22]	Plasma	1g every 8h (infused over 4h)	13
Imipenem [23]	Plasma	1g every 6h (infused over 1h)	12.5
Aztreonam [24]	Plasma	8g every 24h (infused over 24h)	24
Piperacillin/ tazobactam [25]	Plasma	4.5g every 6h (infused over 4h)	35/7

Abbreviations used in Table 1: ELF = epithelial lining fluid

### Data Collection and Outcome Measures

All data were extracted from patients’ inpatient charts and electronic medical records and documented in a structured form. Baseline characteristics collected included basic demographics, length of stay, presence of co-morbidities, APACHE II score at time of XDR-GNB isolation, history of intensive care unit stay or hospital stay within 3 months of XDR-/PDR-GNB isolation, and history of antibiotic use within 3 months of XDR-/PDR-GNB isolation. Details pertaining to the infection (causative organism, site of infection, date of isolation, *in vitro* susceptibility data and concurrent infections) and antibiotic use (type and duration of antibiotics used) was also collected. The following roles of the *i*ACT service were documented: (i) confirmation of bactericidal activity in the empirically selected regimen (i.e. no change in the empirical regimen) (ii) change in empiric therapy, based on *i*ACT results, or (iii) reduction of number of antibiotics empirically employed in combination, based on *i*ACT results. If the *i*ACT service recommended changes in an empiric therapy, the reasons for the recommendations were investigated. The time from culture isolation to request for *i*ACT service, as well time from request for *i*ACT service to time of provision of preliminary and final recommendations by the *i*ACT service for each case was also documented.

The following patient outcomes were collected: (i) 30-day in-hospital all-cause and infection-related mortality, (ii) clinical response, and (iii) microbiological eradication (assessed only in patients with bloodstream infections as repeat cultures were not routinely taken for other sites). Adverse drugs events potentially attributable to *i*ACT-guided therapy were documented.

### Definitions

XDR and PDR were defined as non-susceptibility to at least one agent in all but two or fewer antibiotic categories and non-susceptibility to all agents in all antimicrobial categories respectively [26]. Bacteriostatic activity was defined as the lack of visible growth in the presence of antibiotics at 24h. Bactericidal activity was defined as ≥99.9% decrease in the colony count on subculture of an organism in the presence of antibiotics compared to initial inoculum [13]. An empirical regimen was defined as any antimicrobial regimen employed after isolation of XDR-/PDR-GNB but prior to *i*ACT-guided therapy, selected by the attending ID physician based on individual preference, anecdotal experience or recommendations from published literature. Infections were defined in accordance to definitions provided by the Centers for Disease Control and Prevention (CDC) [27]. Patients were considered immunocompromised if they had acquired immune deficiency syndrome, were neutropenic (absolute neutrophil count < 500/mm^3^) or transplant patients on immunosuppressive agents.

Thirty-day in-hospital all-cause and infection-related mortality was defined as all-cause and infection-related death, as determined by the ID physician, that occurred 30 days from isolation of XDR-/PDR-GNB. Clinical response was defined as partial or complete resolution of infection symptoms as determined by the ID physician, irrespective of GNB eradication, while therapeutic failure was defined as worsening at any time or no improvement of clinical conditions by day 14 of therapy as stated by the ID physician. Microbiologic eradication was only assessed for bloodstream infections, and was defined as the clearance of XDR-/PDR-GNB in all follow-up blood cultures. Nephrotoxicity was defined as per RIFLE criteria [28].

### Data analysis

Categorical data were presented as numbers and percentages; continuous data were presented as mean ± standard deviation, or median (range), depending on the validity of normality assumption. All data were analyzed using IBM SPSS Statistics 21 (IBM Corp., New York).

## Results

### Study Population

From April 2009 to June 2014, 40 patients received *i*ACT-guided therapy. Of these, three patients were excluded as medical records were not available. Two patients developed two episodes of XDR-/PDR-GNB infection more than 90 days apart and were each analyzed as two different cases. Hence, a total of 39 cases were included.

### Baseline Demographics and Clinical Features

The clinical features of the patients were summarized in Table 2. More than one-third of the cases (14/39, 35.9%) were immunocompromised, with solid and hematological malignancies being the commonest co-morbidity (16/39, 41.0%). Most cases received antimicrobial agents prior to the XDR-/PDR-GNB infection (37/39, 94.9%); of these, carbapenems were most frequently prescribed (29/39, 74.4%). Bloodstream infection was the predominant infection (14/39, 35.9%), followed by pneumonia (12/39, 30.8%). Of the 14 cases of bloodstream infections, six (42.9%) were secondary to pneumonia, three (21.4%) were associated with catheter-related infections and three (21.4%) were secondary to intra-abdominal infections.

**Table 2:** Demographics and Clinical Features of Patients Receiving *in Vitro* Combination Antibiotic Testing Guided Therapy (No. of cases = 39).

Characteristics	Mean ± s.d. or median (range) or n (%)
**Basic Demographics**	
**Mean age ± s.d. (yr.)**	56.3 ± 18.3
**Gender**	
**Male**	27 (69.2)
**Female**	12 (30.8)
**Median days of stay (range)**	54 (10–363)
**Past Medical History**	
**Hospitalisation within 3 months**	31 (79.5)
**Intensive care unit stay within 3 months**	22 (56.4)
**Invasive intervention within 3 months**	29 (74.4)
**XDR or PDR-GNB infection within 3 months**	16 (41.0)
**Antibiotic use within 3 months**	37 (94.9)
- Broad-spectrum beta-lactams	14 (35.9)
- 3^rd^ and 4^th^ generation cephalosporins	14 (35.9)
- Carbapenems	29 (74.4)
- Fluoroquinolones	23 (59.0)
- Polymyxins (B or E)	11 (28.2)
**Co-morbidities**	
**No comorbidities**	4 (10.3)
**Ischemic heart disease**	6 (15.4)
**Congestive heart failure**	6 (15.4)
**Chronic kidney disease**	9 (23.1)
**Type II diabetes mellitus**	15 (38.5)
**Hepatic disease**	5 (12.8)
**Solid and hematological malignancy**	16 (41.0)
**Immunocompromised status**	14 (35.9)
**Median Charlson’s comorbidity index (range)**	5 (0–14)
**Type of Infection**	
**Bloodstream infection**	14 (35.9)
- Secondary to respiratory source	6 (15.4)
- Secondary to line infection	3 (7.7)
- Secondary to complicated intra-abdominal infection	3 (7.7)
- Other bloodstream infections ^a^	2 (15.4)
**Hospital/ ventilator-associated pneumonia**	12 (30.8)
**Complicated intra-abdominal infection**	3 (7.7)
**Osteomyelitis**	5 (12.8)
**Others Infections** ^**b**^	5 (12.8)
**Patients with concurrent infections**	10 (25.6)
**Median APACHE II score at time of infection (range)**	13 (4–31)

^a^ Other causes of bloodstream infections include, skin and soft tissue infections (n = 1), and complicated urinary tract infection (n = 1).

^b^ Other infections include complicated urinary tract infection (n = 2), skin and soft tissue infections (n = 2) and mastoiditis (n = 1)

Abbreviations used in Table 2: APACHE II = Acute Physiology and Chronic Health Evaluation II, GNB = Gram-negative bacteria, PDR = pan-drug resistant, XDR = extensively-drug resistant

### The XDR- or PDR-GNB pathogens

Table 3 detailed the resistance mechanisms and *in vitro* susceptibilities of the pathogens. Of the 39 isolates (20 *P*. *aeruginosa*, 13 *A*. *baumannii* and 6 *K*. *pneumoniae*), 31 (79.5%) were XDR and 8 (20.5%) were PDR (four *P*. *aeruginosa*, three *A*. *baumannii* and one *K*. *pneumoniae*). Of the 31 XDR isolates, 19 (61.3%), were sensitive to polymyxin B only. All *A*. *baumannii* harbored *bla*_OXA23_ and *bla*_OXA51_ carbapenemase genes, while 60% (12/20) of the *P*. *aeruginosa* isolates harbored genes encoding MBLs (3 *bla*_VIM_, 9 *bla*_IMP_). A large variety of mechanisms mediating carbapenem resistance was observed for *K*. *pneumoniae* isolates. Amongst the six *K*. *pneumoniae* isolates, four (66.7%) *K*. *pneumoniae* isolates harbored both genes encoding NDM as well as OXA-181 carbapenemases. Of these, one isolate also demonstrated reduction in porin gene expression, while another had presence of efflux pump as determined by phenotypic methods.

**Table 3:** Description of *In Vitro* Antimicrobial Susceptibility and MIC ranges of XDR- or PDR-GNB (20 Strains of *Pseudomonas aeruginosa*, 13 Strains of *Acinetobacter baumannii* and 6 Strains of *Klebsiella pneumoniae*).

	*P*. *aeruginosa* (n = 20)		*A*. *baumannii* (n = 13)		*K pneumoniae* (n = 6)	
Mechanisms of Resistance	VIM (n = 3)		OXA-23-like & OXA-51-like (n = 13)		ESBLs, NDM & OXA-181 (n = 2)	
					ESBLs, AmpC & OXA-48 (n = 2)	
	IMP (n = 9)				ESBLs, NDM, OXA-181 & efflux pump (n = 1)	
					ESBLs, NDM, OXA-181 & porin loss (n = 1)	
Antimicrobial Agents	No. of Non-susceptible isolates (%)	MIC range (mg/L)	No. of Non-susceptible isolates (%)	MIC range (mg/L)	No. of Non-susceptible isolates (%)	MIC range (mg/L)
**Ampicillin/sulbactam**	ND	ND	13 (100)	32/16–≥128/64	6 (100)	≥128/64
**Piperacillin/tazobactam**	20 (100)	32/4–≥256/4	13 (100)	≥256/4	6 (100)	≥256/4
**Cefepime**	20 (100)	32–≥256	13 (100)	≥64	6 (100)	≥64
**Imipenem**	19 (95.0)	2–≥64	13 (100)	32–≥64	6 (100)	4–≥64
**Meropenem**	20 (100)	4–≥64	13 (100)	32–≥64	6 (100)	4–≥64
**Doripenem**	20 (100)	4–≥64	13 (100)	16–≥64	6 (100)	4–≥64
**Aztreonam**	18 (90.0)	8–≥128	NA^a^	16–≥64	6 (100)	≥128
**Levofloxacin**	20 (100)	32–≥64	13 (100)	8–≥64	6 (100)	8–≥64
**Tigecycline**	ND	ND	10 (76.9)^b^	0.5–32	4 (66.7)^b^	0.5–≥64
**Rifampicin**	ND	ND	NA^a^	2–≥64	NA^a^	8–≥64
**Gentamicin**	19 (95.0)	4–≥64	13 (100)	≥64	5 (83.3)	0.5–≥64
**Amikacin**	16 (80.0)	1–≥128	13 (100)	≥128	5 (83.3)	2–≥64
**Polymyxin B**	4 (20.0)	1–8	3 (23.1)	0.5–8	NA^a^	0.5–64

^a^ No breakpoints recommended by Clinical and Laboratory Standards Institute (CLSI)

^b^ Susceptibility defined as ≤2mg/L, according to the Food and Drug Administration (FDA) breakpoints for Enterobacteriaceae

Abbreviations used in Table 3: GNB = Gram negative bacteria, I = intermediate, MIC = minimum inhibitory concentration, NA = not applicable, ND = not done, PDR = pan-drug resistant, R = resistant, XDR = extensively-drug resistant

### Antibiotic Combinations and Role of the *i*ACT Combination Testing Service

A delay between the time of culture isolation and request for *i*ACT service (median: 4 days; range: 2–47 days) was observed. This delay was attributed to the fact that several ID physicians elected to use empirically selected combinations first upon culture isolation, and only requested for *i*ACT service after the patients failed to respond to the empirically selected combinations. Upon request and isolate receipt, the *i*ACT service provided all preliminary results (based on well turbidity) to the ID physicians within 24h. All final *i*ACT recommendations were provided to the ID physicians within 48h.

Prior to *i*ACT-guided therapy, most cases were empirically prescribed combination therapy (35/59, 89.7%) (Table 4). More than half (21/39, 53.8%) of the cases were prescribed three or more antibiotics in combination empirically. Against *P*. *aeruginosa*, polymyxin B plus a carbapenem plus fluoroquinolones or aminoglycosides were the most common three-antibiotic regimen prescribed empirically, while against *A*. *baumannii*, polymyxin B plus a carbapenem plus tigecycline or rifampicin were the most common three-antibiotic regimen prescribed empirically. Empiric therapy was changed by the *i*ACT service in 21 (53.9%) cases (Table 5)–the commonest reasons for change was that empiric antibiotic regimens were not bactericidal *in vitro* (14/21, 66.7%), or that a more appropriate combination regimen, based on the probabilities of PK/PD target attainment at infected sites, could be used (4/21, 19.0%). In 7 (17.9%) patients, the number of antibiotics used in combination empirically was reduced. The *i*ACT service recommended dose changes in 8 (20.5%) patients; most recommendations involved increasing doses or prolonging infusion time to optimize probability of PK/PD target attainment.

**Table 4:** Summary of Antibiotic Therapy Prescribed (a) Empirically, and (b) Based on Recommendations from the *i*ACT Service.

**Antibiotic Therapy prescribed Empirically (%)**^**a**^	**All organisms (n = 39)**	***P*. *aeruginosa* (n = 20)**	***A*. *baumannii* (n = 13)**	***K*. *pneumoniae* (n = 6)**
**Antibiotic monotherapy**	4 (10.3)	3 (15.0)	1 (7.7)	0 (0)
**Two-antibiotic in combination**	14 (35.9)	7 (35.0)	5 (38.5)	2 (33.3)
- Polymyxin B + carbapenem	5 (12.8)	4 (20.0)	0 (0)	1 (16.7)
- Other polymyxin B-based two-antibiotic combinations	6 (15.4)	2 (10.0)	4 (30.8)	1 (16.7)
- Non-polymyxin B based two-antibiotic combinations	3 (7.7)	1 (5.0)	1 (7.7)	0 (0)
**Three antibiotics in combination**	16 (41.0)	8 (40.0)	5 (38.5)	3 (50.0)
- Polymyxin B-based three-antibiotic combinations	14 (35.9)	7 (35.0)	4 (30.8)	3 (50.0)
- Non-polymyxin B based three-antibiotic combinations	2 (5.1)	1 (5.0)	1 (7.7)	0 (0)
**≥Four antibiotics in combination**	5 (12.8)	2 (10.0)	2 (15.4)	1 (16.7)
**Antibiotic Therapy prescribed based on Recommendations from the *i*ACT Service (%)**	**All organisms (n = 39)**	***P*. *aeruginosa* (n = 20)**	***A*. *baumannii* (n = 13)**	***K*. *pneumoniae* (n = 6)**
**Two-antibiotic in combination**	24 (61.5)	11 (55.0)	10 (76.9)	3 (50.0)
- Polymyxin B + carbapenem	10 (25.6)	2 (10.0)	7 (53.8)	1 (16.7)
- Polymyxin B + rifampicin	1 (2.6)	0 (0)	1 (7.7)	0 (0)
- Polymyxin B + tigecycline	1 (2.6)	0 (0)	1 (7.7)	0 (0)
- Other polymyxin B based two-antibiotic combinations	7 (17.9)	5 (25.0)	0 (0)	2 (33.3)
- Non-polymyxin B two-antibiotic combinations	5 (12.8)	4 (20.0)	1 (7.7)	0 (0)
**Three antibiotics in combination**	15 (38.5)	9 (45.0)	3 (23.1)	3 (50.0)
- Polymyxin B-based three-antibiotic combinations	15 (38.5)	9 (45.0)	3 (23.1)	3 (50.0)

^a^ Antibiotic therapy prescribed by the attending physician prior *to in vitro* combination testing were based on personal preference, physician’s past experience or published literature

Abbreviations used in Table 4: *i*ACT = *in vitro* antibiotic combination testing

**Table 5:** Role of Prospective *i*ACT Service in Guiding Antibiotic Combination Therapy.

Role of *i*ACT Service	Role of Prospective *i*ACT Service (%)
**Confirmation of empiric therapy**	11 (28.2)
**Change in empiric therapy**	21 (53.9)
- Initial antibiotic regimen not bactericidal *in vitro*	14 (35.9)
- Need for polymyxin-sparing regimen	3 (7.7)
- Optimize therapy based on PK/PD	4 (10.3)
**Reduction of no. of antibiotics used empirically in combination**	7 (17.9)
**Change in doses or dosing regimen**	8 (20.5)

Abbreviations used in Table 4: *i*ACT = *in vitro* antibiotic combination testing, PK/PD = pharmacokinetic and pharmacodynamic parameters

Polymyxin B-based two- or three-drug combinations were the commonest *i*ACT-guided therapy prescribed (33/39, 84.6%). Nebulized colistin was initiated in all patients with pneumonia who were prescribed intravenous polymyxin B (16/39, 41.0%). The most common combination recommended against *A*. *baumannii* infections were polymyxin B plus a carbapenem (7/13, 53.8%). Against the *P*. *aeruginosa* isolates, polymyxin B plus carbapenem plus aminoglycosides were most commonly recommended (5/20, 25.0%). Antibiotic combination regimens recommended for *K*. *pneumoniae* infections were highly strain-specific; no single regimen was universally effective against all *K*. *pneumoniae* strains.

### Clinical and Microbiological Outcomes

The outcomes according to infection type and responsible pathogen are detailed in Table 6. Six (15.4%) cases died due to infection; of these, five were critically ill at the time of initiation of *i*ACT-guided therapy (APACHE II score at time of *i*ACT-guided therapy ranged from 17–30 in these patients). For the remaining patient, the initial episode of ventilator-associated pneumonia resolved upon *i*ACT-guided therapy; however, the patient developed a new episode of sepsis within 30 days and died. Clinical response (cure or improvement) was observed in 32 (82.1%) patients. Of note, clinical response was observed for all seven patients in whom the number of antibiotics used in combination was reduced. Out of the 14 patients with bloodstream infections, 11 (78.6%) had documented microbiological eradication. Thirteen (33.3%) patients developed adverse drugs events: 10 (25.6%) developed nephrotoxicity likely secondary to polymyxin B (n = 8) or polymyxin B plus amikacin (n = 2), two (5.1%) developed non-*Clostridium difficile*-associated-diarrhea, and one (2.6%) developed confusion secondary to levofloxacin. No death related to the adverse events was observed. Adverse events resolved in all patients who survived (7/10, 70.0%).

**Table 6:** 30-day In-Hospital All-Cause and Infection-Related Mortality, Clinical Response, and Microbiological Eradication Associated with the Type of Infections and Responsible Pathogens.

Type of infection or pathogen responsible	No. with outcome of interest/ total^a^ (%)	No. without outcome of interest/ total^a^ (%)
**30-day in-hospital all-cause mortality**		
**All infections and pathogens**	8/39 (20.5)	31/39 (79.5)
**Based on type of infections**		
- Hospital/ ventilator-associated pneumonia	4/12 (33.3)	8/12 (66.7)
- Bloodstream infections	3/14 (21.4)	11/14 (78.6)
- Complicated intra-abdominal infections	0/3 (0)	3/3 (100)
- Osteomyelitis	0/5 (0)	5/5 (100)
- Other infections^b^	1/5 (20.0)	4/5 (80.0)
**Based on infecting pathogen:**		
*- P*. *aeruginosa*	5/20 (25.0)	15/20 (75.0)
*- A*. *baumannii*	3/13 (23.1)	10/13 (76.9)
*- K*. *pneumoniae*	0/6 (0)	6/6 (100)
**30-day in-hospital infection-related mortality**		
**All infections and pathogens**	6/39 (15.4)	33/39 (84.6)
**Type of infections**		
- Hospital/ ventilator-associated pneumonia	4/12 (33.3)	8/12 (66.7)
- Bloodstream infections	1/14 (7.1)	13/14 (92.9)
- Complicated intra-abdominal infections	0/3 (0)	3/3 (100)
- Osteomyelitis	0/5 (0)	5/5 (100)
- Other infections^b^	1/5 (20.0)	4/5 (80.0)
**Pathogen responsible**		
*- P*. *aeruginosa*	4/20 (20.0)	16/20 (80.0)
*- A*. *baumannii*	2/13 (15.4)	11/13 (84.6)
*- K*. *pneumoniae*	0/6 (0)	6/6 (0)
**Clinical response at end of therapy**		
**All infections and pathogens**	**32/39 (82.1)**	**7/39 (17.9)**
**Type of infections**		
- Hospital/ ventilator-associated pneumonia	8/12 (66.7)	4/12 (33.3)
- Bloodstream infections	12/14 (85.7)	2/14 (14.3)
- Complicated intra-abdominal infections	3/3 (100)	0/3 (0)
- Osteomyelitis	5/5 (100)	0/5 (0)
- Other infections^b^	4/5 (80.0)	1/5 (20.0)
**Pathogen responsible**		
*- P*. *aeruginosa*	16/20 (80.0)	4/20 (25.0)
*- A*. *baumannii*	11/13 (84.6)	2/13 (15.4)
*- K*. *pneumoniae*	6/6 (100)	0/6 (0)
**Microbiological eradication at end of therapy**^**c**^		
**Type of infections**		
- Bloodstream infections	11/14 (78.6)	3/14 (21.4)
**Pathogen responsible**		
*- P*. *aeruginosa*	3/5 (60.0)	2/5 (40.0)
*- A*. *baumannii*	5/6 (83.3)	1/6 (16.7)
*- K*. *pneumoniae*	3/3 (100)	0/3 (0)

^a^ The denominator reflects the number of patients with the stated infection or infecting pathogen.

^b^ Other infections include complicated urinary tract infection, skin and soft tissue infections and mastoiditis.

^c^ Microbiological eradication was only assessed for patients with bloodstream infections (n = 14).

## Discussion

This study explored the role and feasibility of a prospective *in vitro* combination testing service in our local setting. We found that the *i*ACT service could provide individualized recommendations for the most optimal combination within a rapid turn-around time of 48h and provided an attractive alternative to selecting antibiotic combinations merely based on individual physician’s anecdotal experience. In addition, we observed promising rates of clinical response and infection-related mortality in patients prescribed *i*ACT-guided combination therapy.

The emergence of XDR-GNB worldwide and in Singapore has caused several paradigm shifts in antibiotic therapy [1]. In addition to the revival of old antibiotics, combination therapy has been increasingly accepted as common practice in the treatment of XDR-GNB infections [6]. Unfortunately, choosing an effective combination can be difficult due to several reasons. Traditional single-antibiotic susceptibility results have limited utility in the guiding the selection of combination therapy against XDR-/PDR-GNB. As a result, the vast majority of combinations can only be chosen empirically, based on individual’s physician’s preference [7]. However, selecting a combination based on anecdotal experience is often counter-productive in our local setting, as resistance is often mediated by more than one mechanism, resulting in strain-specific differences in combinations that are bactericidal, even within a single species. Hence, selecting combinations based merely on anecdotal experience can result in the prescription of an ineffective combination, potentially compromising patient outcomes. In addition, such irrational use of combination antibiotics may perpetuate the cycle of increasing resistance, as well as subject the patients to increased risk of adverse events [7].

The prospective *i*ACT service was developed to guide physicians in managing patients with XDR-/PDR-GNB infections. The service employed a bench-to-bedside approach to design rationally optimized and individualized antibiotic combination regimens based on PK and PD principles. An *in vitro* combination testing method, designed to systematically test 80 two- and three-drug combinations within 48h, was first employed to prospectively determine the bactericidal activity of antibiotic combinations *in vitro* against individual strains [13]. Leveraging on the *in vitro* results, clinical ID pharmacists then selected the most appropriate antibiotic combination, taking into consideration all relevant PK parameters, such as the patient’s comorbidity status, patient’s renal and hepatic function and distribution of the antibiotics to the site of infection. Recommendations made by the ID pharmacists do not merely consist of specific antibiotic combinations; instead, individualized dosage regimens (doses, route and infusion time), optimized to achieve PK/PD targets at the site of infection, were recommended to best maximize efficacy and decrease risk of resistance emergence.

In this study, we observed that most physicians empirically prescribed combination therapy prior to *i*ACT-guided therapy, selected based on previously published literature or based on individual physicians’ past experience [9, 29]. Unfortunately, of these, less than half of these empirically prescribed combinations were found to be bactericidal *in vitro*, highlighting the inadequacy of selecting combinations based on anecdotal experience in our local setting. Considerable delay was noted between isolation of XDR-GNB and request for *i*ACT service. This is mainly attributable to the fact that the *i*ACT service was not mandatory; as such, several physicians opted to request for *i*ACT service only after patients failed to respond to the initial empiric combination. This can result in the potential benefits conferred by the *i*ACT-guided combination therapy being negated by the delay in time to appropriate therapy, especially in the critically-ill patients. Moving forward, continued education will be required to reinforce the importance of early referral to the *i*ACT service, to ensure the timely prescription of the most appropriate antibiotic combinations.

Our *i*ACT service employed an *in vitro* combination testing method to guide the selection of antibiotic combinations against each XDR-GNB strain. This can reveal any increased or decreased killing brought about by potential synergy (e.g. mechanistic or subpopulation synergy) or antagonism respectively when two or more antibiotics are combined [30]. In addition to guiding the selection of individualized and rationally optimized combinations in patients with XDR-GNB infections, we found that the *i*ACT service potentially provided other functionalities. In 20% of the patients, the *i*ACT service reduced the number of antibiotics initially used in combination. This suggests that the *i*ACT service plays a role in antimicrobial stewardship, reducing unnecessary or indiscreet antibiotic use.

Overall, we observed an all-cause mortality rate of 21% in our study patients, which compared favorably to mortality rates reported in other studies. In a study by Crusio *et al*, all-cause in-hospital mortality was 47% in patients infected with carbapenem-resistant GNB and treated with polymyxin B-based combinations [31]. Similar rates of 30-day all-cause mortality (43%) were observed in study by Durante-Mangoni *et al* [32]. We acknowledged that the observed differences in observed mortality rates may be contributed by a multitude of factors, such as differences in severity of illness. However, it must be highlighted that the combinations employed for patients in these previously published studies were selected based on anecdotal experience or based on results from previously published studies, and not based on strain-specific *in vitro* combination testing results as per our study. More than 80% of patients receiving *i*ACT guided combination therapy had documented clinical response in our study. Of note, clinical response was observed for all patients, in whom the number of combination antibiotics used empirically was reduced, suggesting that reducing the number of antibiotics in combination based on *i*ACT did not compromise patients’ outcomes.

To date, only one study explored the use of prospective combination testing to guide therapy in non-cystic fibrosis patients. In a case series by Nakamura *et al*, a break-point checkerboard plate technique was employed to guide combination selection against multi-drug resistant *P*. *aeruginosa* [33]. The study, however, selected combinations based solely on the results of *in vitro* testing, and did not take into account patient’s PK/PD parameters. Furthermore, the study only reported patients with clinical improvement. In contrast, our study assessed all patients whose antibiotic regimen was prospectively guided by *i*ACT service; in addition to *in vitro* testing, PK/PD principles were applied as the basis for rational selection of antibiotic combinations and dosing regimens.

Despite our promising findings, this study had several limitations. While we observed promising rates of clinical success and infection-related mortality in our study, we acknowledged that the study was not designed to examine outcomes, as no control group was employed for outcome comparison. Hence, no conclusion could be made with regards to the impact of the *i*ACT service on patient outcomes. Moving forward, a randomized trial will be required to fully evaluate the utility of the service in improving patient outcomes. In addition, given that the *i*ACT service was not mandatory, physicians may be inclined to request *i*ACT service only for certain subset of patients with XDR-GNB infections (e.g. those who did not respond to the initial empiric therapy), which can result in selection bias.

## Conclusion

We found that the prospective *i*ACT service can be feasibly implemented to guide the timely selection of rationally optimized combination regimens against XDRGNB, and played an additional role in reducing indiscreet antibiotic use. While further randomized trials will be required to fully evaluate the utility of the service, it is hoped that the prospective *i*ACT service can be continually employed to guide the rational selection of antibiotic combinations, and to reduce indiscreet use of antibiotic combinations.

## References

[1] Boucher HW, Talbot GH, Bradley JS, Edwards JE, Gilbert D, Rice LB, et al Bad bugs, no drugs: no ESKAPE! An update from the Infectious Diseases Society of America. Clin Infect Dis. 2009;48(1):1–12. Epub 2008/11/28. 10.1086/595011 . 19035777

[2] Ng E, Earnest A, Lye DC, Ling ML, Ding Y, Hsu LY. The excess financial burden of multidrug resistance in severe gram-negative infections in Singaporean hospitals. Ann Acad Med Singapore. 2012;41(5):189–93. Epub 2012/07/05. . 22760715

[3] Patel SJ, Oliveira AP, Zhou JJ, Alba L, Furuya EY, Weisenberg SA, et al Risk factors and outcomes of infections caused by extremely drug-resistant gram-negative bacilli in patients hospitalized in intensive care units. American journal of infection control. 2014;42(6):626–31. 10.1016/j.ajic.2014.01.027 24725516 PMC4083852

[4] Zavascki AP, Goldani LZ, Li J, Nation RL. Polymyxin B for the treatment of multidrug-resistant pathogens: a critical review. J Antimicrob Chemother. 2007;60(6):1206–15. 10.1093/jac/dkm357 . 17878146

[5] Kollef MH, Golan Y, Micek ST, Shorr AF, Restrepo MI. Appraising contemporary strategies to combat multidrug resistant gram-negative bacterial infections—proceedings and data from the Gram-Negative Resistance Summit. Clin Infect Dis. 2011;53 Suppl 2:S33–55; quiz S6-8. 10.1093/cid/cir475 21868447 PMC3161517

[6] Ahmed A, Azim A, Gurjar M, Baronia AK. Current concepts in combination antibiotic therapy for critically ill patients. Indian journal of critical care medicine: peer-reviewed, official publication of Indian Society of Critical Care Medicine. 2014;18(5):310–4. 10.4103/0972-5229.132495 24914260 PMC4047693

[7] Zavascki AP, Bulitta JB, Landersdorfer CB. Combination therapy for carbapenem-resistant Gram-negative bacteria. Expert Rev Anti Infect Ther. 2013;11(12):1333–53. Epub 2013/11/07. 10.1586/14787210.2013.845523 . 24191943

[8] Kitchel B, Rasheed JK, Endimiani A, Hujer AM, Anderson KF, Bonomo RA, et al Genetic factors associated with elevated carbapenem resistance in KPC-producing Klebsiella pneumoniae. Antimicrobial agents and chemotherapy. 2010;54(10):4201–7. 10.1128/AAC.00008-10 20660684 PMC2944623

[9] Lim TP, Tan TY, Lee W, Sasikala S, Tan TT, Hsu LY, et al In-vitro activity of polymyxin B, rifampicin, tigecycline alone and in combination against carbapenem-resistant Acinetobacter baumannii in Singapore. PloS one. 2011;6(4):e18485 10.1371/journal.pone.0018485 21533030 PMC3080872

[10] Voets GM, Fluit AC, Scharringa J, Cohen Stuart J, Leverstein-van Hall MA. A set of multiplex PCRs for genotypic detection of extended-spectrum beta-lactamases, carbapenemases, plasmid-mediated AmpC beta-lactamases and OXA beta-lactamases. International journal of antimicrobial agents. 2011;37(4):356–9. 10.1016/j.ijantimicag.2011.01.005 . 21353487

[11] Teo J, Ngan G, Balm M, Jureen R, Krishnan P, Lin R. Molecular characterization of NDM-1 producing Enterobacteriaceae isolates in Singapore hospitals. Western Pacific surveillance and response journal: WPSAR. 2012;3(1):19–24. 10.5365/WPSAR.2011.2.4.010 23908903 PMC3729072

[12] Roy S, Datta S, Viswanathan R, Singh AK, Basu S. Tigecycline susceptibility in Klebsiella pneumoniae and Escherichia coli causing neonatal septicaemia (2007–10) and role of an efflux pump in tigecycline non-susceptibility. The Journal of antimicrobial chemotherapy. 2013;68(5):1036–42. 10.1093/jac/dks535 . 23335112

[13] Aaron SD, Ferris W, Henry DA, Speert DP, Macdonald NE. Multiple combination bactericidal antibiotic testing for patients with cystic fibrosis infected with Burkholderia cepacia. Am J Respir Crit Care Med. 2000;161(4 Pt 1):1206–12. Epub 2000/04/14. 10.1164/ajrccm.161.4.9907147 . 10764313

[14] Tod M, Lortholary O, Seytre D, Semaoun R, Uzzan B, Guillevin L, et al Population pharmacokinetic study of amikacin administered once or twice daily to febrile, severely neutropenic adults. Antimicrob Agents Chemother. 1998;42(4):849–56. Epub 1998/04/29. . 9559795 10.1128/aac.42.4.849 PMC105554

[15] Rebuck JA, Fish DN, Abraham E. Pharmacokinetics of intravenous and oral levofloxacin in critically ill adults in a medical intensive care unit. Pharmacotherapy. 2002;22(10):1216–25. . 12389872 10.1592/phco.22.15.1216.33484

[16] Conte JE Jr, Golden JA, McIver M, Little E, Zurlinden E. Intrapulmonary pharmacodynamics of high-dose levofloxacin in subjects with chronic bronchitis or chronic obstructive pulmonary disease. Int J Antimicrob Agents. 2007;30(5):422–7. 10.1016/j.ijantimicag.2007.05.023 . 17716873

[17] Gumbo T, Louie A, Deziel MR, Liu W, Parsons LM, Salfinger M, et al Concentration-dependent Mycobacterium tuberculosis killing and prevention of resistance by rifampin. Antimicrob Agents Chemother. 2007;51(11):3781–8. Epub 2007/08/29. AAC.01533-06 [pii] 10.1128/AAC.01533-06 . 17724157 PMC2151424

[18] Kwa AL, Lim TP, Low JG, Hou J, Kurup A, Prince RA, et al Pharmacokinetics of polymyxin B1 in patients with multidrug-resistant Gram-negative bacterial infections. Diagnostic microbiology and infectious disease. 2008;60(2):163–7. 10.1016/j.diagmicrobio.2007.08.008 . 17916420

[19] Rodvold KA, Gotfried MH, Cwik M, Korth-Bradley JM, Dukart G, Ellis-Grosse EJ. Serum, tissue and body fluid concentrations of tigecycline after a single 100 mg dose. J Antimicrob Chemother. 2006;58(6):1221–9. Epub 2006/10/03. dkl403 [pii] 10.1093/jac/dkl403 . 17012300

[20] Tam VH, McKinnon PS, Akins RL, Drusano GL, Rybak MJ. Pharmacokinetics and pharmacodynamics of cefepime in patients with various degrees of renal function. Antimicrob Agents Chemother. 2003;47(6):1853–61. Epub 2003/05/23. 12760858 10.1128/AAC.47.6.1853-1861.2003 PMC155813

[21] Jaruratanasirikul S, Sriwiriyajan S, Punyo J. Comparison of the pharmacodynamics of meropenem in patients with ventilator-associated pneumonia following administration by 3-hour infusion or bolus injection. Antimicrob Agents Chemother. 2005;49(4):1337–9. Epub 2005/03/29. 49/4/1337 [pii] 10.1128/AAC.49.4.1337-1339.2005 . 15793108 PMC1068632

[22] Jaruratanasirikul S, Wongpoowarak W, Kositpantawong N, Aeinlang N, Jullangkoon M. Pharmacodynamics of doripenem in critically ill patients with ventilator-associated Gram-negative bacilli pneumonia. Int J Antimicrob Agents. 2012;40(5):434–9. 10.1016/j.ijantimicag.2012.07.014 . 22959555

[23] Sakka SG, Glauner AK, Bulitta JB, Kinzig-Schippers M, Pfister W, Drusano GL, et al Population pharmacokinetics and pharmacodynamics of continuous versus short-term infusion of imipenem-cilastatin in critically ill patients in a randomized, controlled trial. Antimicrob Agents Chemother. 2007;51(9):3304–10. 10.1128/AAC.01318-06 17620371 PMC2043189

[24] LaPlante KL, Sakoulas G. Evaluating aztreonam and ceftazidime pharmacodynamics with Escherichia coli in combination with daptomycin, linezolid, or vancomycin in an in vitro pharmacodynamic model. Antimicrobial agents and chemotherapy. 2009;53(10):4549–55. 10.1128/AAC.00180-09 19620335 PMC2764192

[25] Shea KM, Cheatham SC, Wack MF, Smith DW, Sowinski KM, Kays MB. Steady-state pharmacokinetics and pharmacodynamics of piperacillin/tazobactam administered by prolonged infusion in hospitalised patients. Int J Antimicrob Agents. 2009;34(5):429–33. 10.1016/j.ijantimicag.2009.07.004 . 19726163

[26] Magiorakos AP, Srinivasan A, Carey RB, Carmeli Y, Falagas ME, Giske CG, et al Multidrug-resistant, extensively drug-resistant and pandrug-resistant bacteria: an international expert proposal for interim standard definitions for acquired resistance. Clinical microbiology and infection: the official publication of the European Society of Clinical Microbiology and Infectious Diseases. 2012;18(3):268–81. 10.1111/j.1469-0691.2011.03570.x . 21793988

[27] Horan TC, Andrus M, Dudeck MA. CDC/NHSN surveillance definition of health care-associated infection and criteria for specific types of infections in the acute care setting. American journal of infection control. 2008;36(5):309–32. 10.1016/j.ajic.2008.03.002 . 18538699

[28] Kellum JA, Bellomo R, Ronco C. Definition and classification of acute kidney injury. Nephron Clinical practice. 2008;109(4):c182–7. 10.1159/000142926 . 18802365

[29] Lim TP, Lee W, Tan TY, Sasikala S, Teo J, Hsu LY, et al Effective antibiotics in combination against extreme drug-resistant Pseudomonas aeruginosa with decreased susceptibility to polymyxin B. PloS one. 2011;6(12):e28177 10.1371/journal.pone.0028177 22162759 PMC3230594

[30] Yahav D, Farbman L, Leibovici L, Paul M. Colistin: new lessons on an old antibiotic. Clinical microbiology and infection: the official publication of the European Society of Clinical Microbiology and Infectious Diseases. 2012;18(1):18–29. 10.1111/j.1469-0691.2011.03734.x . 22168320

[31] Crusio R, Rao S, Changawala N, Paul V, Tiu C, van Ginkel J, et al Epidemiology and outcome of infections with carbapenem-resistant Gram-negative bacteria treated with polymyxin B-based combination therapy. Scandinavian journal of infectious diseases. 2014;46(1):1–8. 10.3109/00365548.2013.844350 . 24206450

[32] Durante-Mangoni E, Signoriello G, Andini R, Mattei A, De Cristoforo M, Murino P, et al Colistin and rifampicin compared with colistin alone for the treatment of serious infections due to extensively drug-resistant Acinetobacter baumannii: a multicenter, randomized clinical trial. Clin Infect Dis. 2013;57(3):349–58. 10.1093/cid/cit253 . 23616495

[33] Nakamura I, Yamaguchi T, Tsukimori A, Sato A, Fukushima S, Mizuno Y, et al Effectiveness of antibiotic combination therapy as evaluated by the Break-point Checkerboard Plate method for multidrug-resistant Pseudomonas aeruginosa in clinical use. Journal of infection and chemotherapy: official journal of the Japan Society of Chemotherapy. 2014;20(4):266–9. 10.1016/j.jiac.2013.12.005 . 24486172

